# Mobile Phone Base Stations: Eltiti et al. Respond

**DOI:** 10.1289/ehp.10733R

**Published:** 2008-02

**Authors:** Stacy Eltiti, Denise Wallace, Anna Ridgewell, Riccardo Russo, Elaine Fox

**Affiliations:** Biola University, La Mirada, California, E-mail: stacy.eltiti@biola.edu; University of Essex Colchester, Essex, United Kingdom

Three letters have questioned the validity of the conclusions drawn in our recent article on the short-term effects of GSM (global system for mobile communication) and UMTS (universal mobile telecommunications system) base station signals (Eltiti et al. 2007). Most of the concerns are founded in misunderstandings of the study, and we hope to clarify these issues here. We assessed whether people could detect the presence of a 10-mW/m^2^ signal over a 50-min period (not 10 μW as claimed by Zinelis). This level of exposure is roughly equivalent to standing within 60 m of a mobile phone base station and was based on prior scientific evidence (Mann et al. 2000). We also measured a range of variables within three classes of response: physiological response, self-reported well-being, and actual symptoms experienced.

We found no evidence that people could detect the presence of the EMF (electromagnetic field) signal, and Cohen et al.’s assertion that “this conclusion is erroneous” is completely unfounded. Their conclusion arises from a misunderstanding of the receiver operating characteristic (ROC) curve analysis. ROC curves and *d*′ values tell us how accurate participants are in discriminating a signal from a nonsignal. This standard psychophysical measure (*d*′) provides a measure of accuracy independent of bias. Thus, a *d*′ score of 0 means that the proportion of hits (respond “on” when on) is the same as for false alarms (respond “on” when off) and indicates that people are unable to detect a signal (Macmillan and Creelman 2005). In this case, the ROC curve will fall roughly across the graph at a 45° angle, (as we found (Eltiti et al. 2007). As shown in Table 1, both the hits and false alarms were not different from what was expected by chance, and this was true for both the sensitive and the control groups. Thus, the comment by Cohen et al. is unfounded and inaccurate.

We measured the following physiological responses: blood volume pulse, heart rate, blood pressure, and skin conductance response (SCR). The SCR in particular is considered to be one of the most sensitive measures of physiological arousal (Curtin et al. 2007). Although the sensitive group was more aroused at baseline than controls—which has been reported many times before—this physiological arousal was not related to the EMF signal. The hyperarousal of the sensitive group is of interest in its own right, as noted in our article (Eltiti et al. 2007). However, we found no evidence that either GSM or UMTS affected any physiological measure.

In our study (Eltiti et al. 2007), participants were free to report any symptoms they experienced at any time during the testing session. The number of symptoms experienced by the sensitive individuals was not, however, related to the presence of an EMF signal. In his letter, Zinelis argues that our statistical power was too low and the length of exposure too short to allow symptoms to emerge. First, the statistical power (0.75) in our study was actually very high for this field of research. Second, extensive pilot testing and interviews with study participants revealed that the people we tested reported that they usually experience their typical symptoms within minutes of being exposed to EMF signals. The fact that the symptoms were elicited under the open provocation, but not in the double-blind session, provides evidence that these sensitive people experienced a number of unpleasant symptoms, but these were not related to the presence of an EMF signal. Thus, our data (Eltiti et al. 2007) contradict the points raised by Zinelis.

All three letters about our article (Eltiti et al. 2007) question the validity of our conclusions with regard to the subjective well-being measures. We did report a number of effects, two of which remained significant following Bonferroni correction. In their letter, Röösli and Huss question whether we should have used such a statistical correction in the current context. This is indeed an important and debatable issue. However, we believe that we took the most reasonable approach, given the weight of the evidence from the other indicators in our own study as well as from the bulk of other research in this area (e.g., for review, see Rubin et al. 2005). To illustrate, previous research has reported positive (e.g., Hietanen et al. 2002), negative (e.g., Zwamborn et al. 2003), and no effect of short-term EMF exposure on health indices (e.g., Lyskov et al. 2001; Regel et al. 2006; Rubin et al. 2006). Thus, the use of two-tailed tests seems most appropriate. If we apply the Tukey-Ciminera-Heyse correction for highly correlated end points, as suggested by Röösli and Huss, we are left with a significant difference in self-reported anxiety [*t* (43) = 2.89; *p* = 0.006] and tension [*t* (43) = 2.94; *p* = 0.005] between the UMTS and sham exposures for the sensitive participants. Also, the magnitude of the effect was very small (< 1 point difference on a 10-point scale). No other differences were significant.

A 2 (group) × 3 (condition) × 6 (exposure order) mixed analysis of variance (ANOVA) for anxiety, tension, and arousal resulted in significant two-way interactions of condition by exposure order for all three visual analogue scales (VAS) [*F*-values (10, 292) > 3.41; *p*-values = 0.001), which did not interact with group [*F*-values (10, 292) < 1.08; *p*-values > 0.05). This two-way interaction is difficult to interpret given the six levels of exposure order. To aid interpretation, we conducted a series of 2 (group) × 3 (condition) × 3 (first exposure) mixed ANOVAs for anxiety, tension, and arousal. This resulted in significant two-way interactions [*F*-values (4, 304) > 5.88; *p*-values = 0.001), but not a three-way interaction [*F*-values (4, 304) < 1.39; *p*-values > 0.05). Again, the first exposure did not interact with group. As shown in Figure 1, the significant differences depended on which condition the participant received first. When the first exposure was GSM, the VAS for GSM were higher than for sham [*t*-values (52) > 3.72; *p*-values = 0.001); the same was found for UMTS [*t*-values (52) > 2.66; *p*-values < 0.01); and sham [*t*-values (51) > 2.12; *p*-values < 0.04). None of the other comparisons were significant (Figure 1). This confirms our previous conclusion that difference in self-reported VAS for anxiety, tension, and arousal is attributable to order effects.

In conclusion, we appreciate the opportunity to discuss the interpretation of data in this controversial area. However, in our view the conclusions drawn in our article are fair and accurate, and we do not think that the letters have raised any issues that would lead us to modify those conclusions. As we made clear in our article (Eltiti et al. 2007), we did examine short-term effects of EMF exposure and therefore can draw no conclusions about the possible long-term effects on human health.

## Figures and Tables

**Figure 1:**
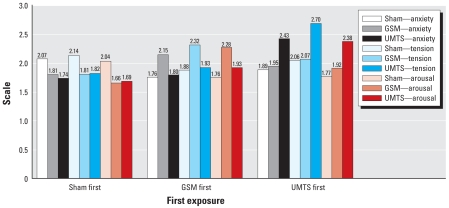
Visual analog scales of anxiety, tension, and arousal by condition by first exposure for all participants.

**Table 1:** *d*′, sensitivity (%), and specificity (%) by exposure duration by group.

	Exposure duration (min)	*d*′	Sensitivity (“on” when on)	Specificity (“off” when off)
Expected value when people do not know the source of the signal			66.6	33.3
Sensitive group	5	−0.08	66.4	32.7
	50	0.20	69.3	40.9
Control group	5	0.10	51.7	50.1
	50	0.06	48.0	54.3
